# The Business of Scientific Publishing

**DOI:** 10.1120/jacmp.v17i1.6239

**Published:** 2016-01-08

Scientific publishing is a major endeavor of the American Association of Physicists in Medicine (AAPM). Although publishing requires substantial resources, it has the potential to generate both reputation and revenue to further the missions of a professional society. The AAPM owns and publishes two journals: *Medical Physics* and the *Journal of Applied Clinical Medical Physics* (*JACMP*), which synergistically span the breadth of its membership's professional endeavors. *Medical Physics* publishes research concerned with the application of physics and mathematics to the solution of problems in medicine and human biology, with an emphasis on theoretical and experimental approaches. The *JACMP* is an applied journal that publishes papers designed to help clinical medical physicists and other health professionals perform their responsibilities more effectively and efficiently for the increased benefit of the patient. The two journals reflect two different missions, engage two different publication approaches, and operate under two different financial models. Nevertheless, the two journals, each with its own identity, are complementary in their mission and scope; in effect, the “new science” of today fostered by *Medical Physics* becomes the “practical clinical science” of tomorrow disseminated by *JACMP*. The purpose of this editorial is to explain the complex interplay of economic factors that impact financial decisions governing the journals and the returns they bring to the AAPM.

The scientific literature represents a compact between the scientific community and society. Articles that become part of “the literature” are held in high regard by the public, with imposed expectations of a robust scientific peer review process; a product that is consistent in format, well organized, free from errors, and visually appealing; accessibility through known and accepted portals (electronic or otherwise) with standard indices that allow for efficient retrieval of specified content; and long‐term persistence through reliable archival approaches. This complex set of requirements has associated costs that are significant.

The budget for *Medical Physics* reflects expenses that include diverse items such as printing and paper, shipping, commissions on advertising sales, copyediting and production, compliance with the open‐access policies of the NIH and other funding sources, editor and editorial office expenses, and AAPM staff support charges. It is important to note that a substantial fraction of Medical Physics expenses is incurred for the express purpose of generating revenue. The revenue side of the 2016 *Medical Physics* budget includes advertising (e.g., printed ads, online banner ads, PDF cover pages), nonmember subscriptions (e.g., libraries, consortia), excess page charges, printed color figure fees, reprint fees, and author‐sponsored open‐access article processing charges. All net revenue from *Medical Physics* remains with the AAPM to fund its broad range of activities that benefit the field of medical physics.

All the activities that the AAPM undertakes in fulfilling its mission to advance the field of medical physics are funded by three main sources: member dues, the annual meeting, and *Medical Physics*. All net revenue from the journals belongs to the AAPM, and any deficits incurred by the journals must be covered by the AAPM. *Medical Physics* has been self‐sustaining for nearly 20 years. AAPM members can find a more detailed discussion of journal finances and membership dues considerations in the November/December 2015 AAPM Newsletter.

*JACMP* is an online‐only, gold open‐access, bimonthly journal that follows a very different publication model. The budget for *JACMP* reflects expenses that include article layout and proofreading, copyediting, metadata markup, the manuscript platform, article hosting, and editor and editorial office expenses. The revenue side of the *JACMP* budget contains two sources of revenue: advertising and the newly instituted $500‐per‐article processing charge. *JACMP* has made important contributions toward advancement of the gold open‐access publishing paradigm. Consistent with the gold open‐access movement, *JACMP* is freely available to all readers. An open‐access journal, however, is not inherently less expensive to publish than a subscription‐based journal; under the usual open‐access paradigm, publication costs are shifted from the readers/subscribers to the authors, who benefit from more widespread access to their work.

Most (if not all) reputable open‐access journals require authors to pay an article processing charge, which typically runs between $1,500–$3,000. While the *JACMP* has allowed authors to publish without fees since its inception 16 years ago, the deficit in *JACMP* publication costs has been funded solely by the AAPM since it acquired the journal in 2012. Recent growth of the *JACMP* has increased the financial burden of publication. Advertising revenue currently covers a relatively small fraction of *JACMP* publication costs, and expanded advertising opportunities are being explored. Consistent with the open‐access paradigm, authors are now assessed an article processing charge of $500 for an article published in *JACMP;* a waiver policy for authors from developing countries is being developed. Advertising and article processing charges are expected to offset roughly two‐thirds of *JACMP* publication costs in 2016, with the AAPM funding the remainder. The goal is to put *JACMP* on a trajectory of financial self‐sufficiency, to ensure the long‐term sustainability of this resource to the community.

*JACMP* is published online only, which avoids all the costs of paper, printing, and delivery; *Medical Physics* was first published on paper long before online publishing was viable, and it is currently published in both paper and online formats. The paper version of *Medical Physics* actually continues to attract enough revenue from subscriptions and advertising to significantly exceed those additional print‐related expenses, so the print version has remained independently viable. During the past two years, however, the print version of *Medical Physics* has experienced a slight decline in revenue. The AAPM constantly monitors the viability of print and seeks to understand any possible negative economic impact of a transition to an online‐only publication; the AAPM is prepared to make such a transition when (and if) a transition would be of net economic benefit. It should be noted that five categories of *Medical Physics* content are already published only online (review articles, task group reports, special focus series articles, PhD thesis abstracts, and book reviews).

The AAPM recently has devoted substantial effort toward defining, integrating, and reaffirming its interests in scientific publishing. An Ad Hoc Committee on Journal Publications was convened between 2013 and 2014. The charge of this committee was to address the scope of the journals; to review business management approaches to maintain quality of content, to maintain a healthy financial position, and to ensure availability to medical physicists throughout the world; and to maintain a process of editorial review that balances speed of publication and quality of review. The final report contains valuable recommendations that are still being evaluated for practical implementation, but two key foundation‐laying initiatives were enacted in 2015. The first initiative was the release of a request for proposals (RFP) to seek a common platform of publishing and manuscript review services for both journals. A common platform will provide a uniform experience for authors and readers of both journals, facilitate efficient exchange of manuscripts between the journals when appropriate, strengthen the AAPM branding of the two journals, and yield an economic benefit to the AAPM. This RFP is under active solicitation. The second initiative was the creation of a single Journals Business Management Committee (JBMC) so that both journals, despite having different financial models, could benefit from a common group of individuals setting policy and providing economic oversight. The unifed JBMC was created as a merger of two previous committees that were responsible for each journal separately. The JBMC is tasked with overseeing the financial aspects of the AAPM's two journals to strategically evolve their financial stability while ensuring the highest level of quality for both. The voting members of the JBMC include the Chair, the AAPM Treasurer, three at‐large members, the Editor‐in‐Chief of *Medical Physics*, and the Editor‐in‐Chief of *JACMP*. The Chair of the JBMC is an ex officio member of the editorial boards of both journals. The JBMC reports to the AAPM's Administrative Council.

Publishing will continue to play an important role in the AAPM mission to advance the science, education, and professional practice of medical physics. The AAPM, therefore, is committed to ensuring that its two scientific journals thrive and flourish. The JBMC is the steward of the journals' collective financial welfare, while the editors, along with the editorial boards, cultivate the scientific quality and integrity of their respective journals. Together, these efforts have nurtured two highly respected journals that make important complementary contributions to our field. The scientific publishing enterprise, however, is undergoing significant transformation, presenting challenges that the JBMC monitors constantly; AAPM publishing strategies must anticipate, assess, and respond in a manner that balances sometimes‐competing interests. Examples of such strategies include the designation of more *Medical Physics* content as open access (including the hybrid gold open‐access option that allows authors, for a fee, to select open‐access status for their article), the integration of a single JBMC for the two journals, the implementation of a common publication platform, and the enhancement of online features for both journals. The ultimate goal has always been the advancement of the journals' status in the literature combined with fulfillment of the AAPM's mission.

Samuel G. Armato III, PhD

Chair, AAPM Journals Business Management Committee

Michael D. Mills, PhD

Editor‐in‐Chief *JACMP*

The AAPM Journals Business Management Committee: Samuel G. Armato III, Chair;

J. Daniel Bourland, Michael D. Mills, Matthew B. Podgorsak, Michelle M. Svatos,

Jeffrey F. Williamson. Non‐Voting Appointments: Bruce H. Curran,

Shiva K. Das, Mitchell M. Goodsitt, Per H. Halvorsen,

Christopher H. Marshall, Timothy D. Solberg

